# Cholesterol Awareness in the Transgender Population: A Comparison With Cisgender Cohort

**DOI:** 10.1016/j.jacadv.2025.102477

**Published:** 2025-12-18

**Authors:** Akash Sharma, Ankit Agrawal, Fnu Shalini, Erin D. Michos

**Affiliations:** a Department of Cardiovascular Medicine, University of Kansas Medical Center, Kansas City, Kansas, USA; b Center for Global Health Research, Saveetha Medical College and Hospital, Saveetha Institute of Medical and Technical Sciences, Saveetha University, Chennai, Tamil Nadu, India; c Department of Cardiovascular Medicine, University of Arkansas for Medical Sciences, Little Rock, Arkansas, USA; d Department of Internal Medicine, University of Buffalo, Buffalo, New York, USA; e Division of Cardiology, Johns Hopkins University School of Medicine, Baltimore, Maryland, USA

**Keywords:** cholesterol awareness, cisgender, transgender

**What is the clinical question being addressed?**

What is the cholesterol awareness among the transgender population?

**What is the main finding?**

A gap exists in cholesterol management among transgender individuals, who were more likely to report having high cholesterol and recent cholesterol checks, but were less likely to report taking cholesterol medications.

Elevated cholesterol levels increase the risk of atherosclerotic cardiovascular disease (ASCVD), which is a leading cause of death. To prevent ASCVD, the U.S. Preventive Services Task Force recommends using statins for primary prevention.^1^ However, social determinants can affect cholesterol levels, with younger age, male sex, lack of insurance, and no access to health care being associated with lower cholesterol screening.^2^ There have been studies regarding cholesterol awareness in the general population, but there is a gap in studies for transgender people.^3^ Therefore, we conducted this study to assess the awareness of cholesterol in the transgender population.

The study adhered to the STROBE (Strengthening the Reporting of Observational Studies in Epidemiology) Checklist, using 2019 and 2021 data from the U.S.'s BRFSS (Behavioral Risk Factor Surveillance System) survey. BRFSS is a comprehensive telephone survey in the United States that collects data on chronic conditions and behavioral patterns, which is self-reported by participants. Institutional Review Board approval was not required since this is a publicly available, deidentified database. Different characteristics of the participants were collected and compared between transgender and cisgender adults. Cholesterol awareness was defined by three self-reported parameters: total cholesterol checked in the last 5 years, high total cholesterol (self-report of doctor’s diagnosis), and intake of cholesterol-lowering medications. The primary outcome was defined as the comparison of the cholesterol awareness between cisgender and transgender populations. Initially, the 1:1 propensity score was calculated by matching income, diabetes, myocardial infarction, stroke, education, age, urban or rural, race, and employment between transgender and cisgender individuals by using the nearest neighborhood method. Logistic regression analysis was then performed with adjustment for propensity scores to calculate with 95%, considering a *P* value <0.05 as statistically significant. All analyses were conducted using R statistical software.

Our study involved 473,443 individuals, with 470,975 being cisgender (215,706 [45.8%] males, and 255,268 [54.2%] females) and 2,468 being transgender persons (1,246 [50.1%] males, and 1,231 [49.9%] females). Table 1 represents the weighted prevalence of the different characteristics of transgender and cisgender individuals. We found that transgender individuals had lower incomes, lower education status, and lower prevalence of cholesterol checks than cisgender individuals. They also had a higher prevalence of depression, poor physical health, and poor general health. We found that they had a lower prevalence of high cholesterol, but a higher percentage of transgender individuals were taking more cholesterol medications with less frequency of cholesterol checks.

**Table 1:** A Comparison of Cisgender and Transgender Population Characteristics (Weighted)

	Cisgender, (n = 250,168,625	Transgender (n = 1,496,798	*P* Value
Income			
<15,000	15,181,338 (6.1)	154,974 (10.4)	
15,000-25000	26,119,310 (10.4)	234,022 (15.6)	
25,000-35000	22,620,425 (9.0)	162,267 (10.8)	
35,000-50000	26,270,796 (10.5)	127,959 (8.5)	
>50,000	82,242,379 (32.9)	336,946 (22.5)	
NA	50,273,013 (20.1)	383,120 (25.6)	
Education			
Did not graduate from high school	28,934,364 (11.6)	313,804 (21.0)	<0.001
Graduated from high school	70,784,532 (28.3)	509,609 (34.0)	
Attended college or technical school	77,856,323 (31.1)	424,927 (28.4)	
Graduated from college or technical school	71,597,460 (28.6)	244,740 (16.4)	
Age (in y)			
18-24	28,020,716 (11.2)	570,803 (38.1)	<0.001
25-34	39,898,227 (15.9)	316,209 (21.1)	
35-44	40,908,863 (16.4)	194,872 (13.0)	
45-54	40,384,859 (16.1)	151,260 (10.1)	
55-64	43,263,009 (17.3)	122,961 (8.2)	
>65	57,692,950 (23.1)	140,692 (9.4)	
Race			
White only, non-Hispanic	162,702,950 (65.0)	886,871 (59.3)	<0.001
Black only, non-Hispanic	30,825,650 (12.3)	133,128 (8.9)	
American Indian or Alaskan Native only	2,569,601 (1.0)	30,455 (2.0)	
Asian only, non-Hispanic	10,392,205 (4.2)	52,160 (3.5)	
Native Hawaiian or other Pacific Islander only	612,167 (0.2)	7,707 (0.5)	
Other race only, non-Hispanic	1,472,219 (0.6)	7,881 (0.5)	
Multiracial, non-Hispanic	3,397,071 (1.4)	34,042.7 (2.3)	
Hispanic	35,979,137 (14.4)	329,126 (22.0)	
NA	2,215,205 (0.9)	15,426 (1.0)	
High cholesterol	74,346,407 (35.6)	301,735 (27.7)	<0.001
Cholesterol check in last 5 y			
Never checked	20,680,566 (8.3)	201,880 (13.5)	<0.001
Not checked in last 5 y	8,805,708 (3.5)	53,077 (3.5)	
Checked in 5 y	201,487,500 (87.2)	1,044,428 (80.4)	
Urban/rural			
Urban	231,150,475 (92.4)	1,400,221 (93.6)	0.163
Rural	18,925,749 (7.6)	95,363 (6.4)	
Taking cholesterol medication	93,643,372 (65.3)	592,753 (78.1)	<0.001
BMI			
Underweight	4,046,816 (1.8)	84,278 (6.4)	<0.001
Normal weight	67,882,172 (30.0)	437,259 (33.4)	
Overweight	78,364,923 (34.6)	320,101 (24.4)	
Obese	76,279,710 (33.7)	468,749 (35.8)	
Hypertension	163,637,921 (65.4)	1,124,516 (75.1)	<0.001
Diabetes	29,008,871 (11.6)	150,314 (10.0)	0.008
Chronic kidney disease	8,080,464 (3.2)	63,470 (4.2)	0.011
Current smokers	24,994,677 (10.4)	169,870 (12.0)	<0.001
Heavy alcohol consumption	14,120,247 (6.0)	99,751 (7.4)	0.102
Have some health insurance	216,595,819 (88.7)	1,221,746 (85.3)	<0.001
Not able to see the doctor because of the cost	29,391,124 (11.7)	389,917 (26.1)	<0.001
General health			
Excellent	43,894,571 (17.5)	203,200 (13.6)	<0.001
Very good	80,838,206 (32.3)	381,477 (25.5)	
Good	80,647,654 (32.2)	478,377 (32.0)	
Fair	33,195,428 (13.3)	318,462 (21.3)	
Poor	10,967,918 (4.4)	109,062 (7.3)	
Depression	49,065,942 (19.6)	713,199 (47.6)	<0.001
Does have a personal family doctor	200,911,051 (80.3)	1,055,313 (70.5)	<0.001
Physical health is not good	84,774,527 (33.9)	746,447 (49.9)	<0.001
Employed	141,136,822 (56.7)	778,768 (52.4)	0.639

Values are n (%).

Prevalences are weighted to account for the complex survey design of BRFSS and to give national representative estimates.

BMI = body mass index; NA = not applicable.

After 1:1 propensity matching, 1,298 transgender people were matched with 1,298 cisgender individuals. Propensity-matched analysis showed that transgender people had higher odds of self-reported cholesterol checks (OR: 1.03; 95% CI: 1.02-1.05; *P* < 0.001) in the past 5 years compared with cisgender individuals. However, they also had higher odds of self-reported high cholesterol levels (OR: 1.12; 95% CI: 1.08-1.16; *P* < 0.001) and lower odds of reporting cholesterol medication use (OR: 0.95; 95% CI: 0.91-0.98; *P* = 0.01). We also examined other determinants of social well-being (such as lack of insurance, inability to afford a doctor, poor general health, poor physical health, and depression) in relation to cholesterol awareness among transgender people. No personal insurance (OR: 1.15; 95% CI: 1.01-1.32; *P* = 0.04), no personal doctor (OR: 1.12; 95% CI: 1.02-1.23; *P* = 0.01), inability to afford a doctor (OR: 1.12; 95% CI: 1.07-1.16; *P* < 0.001), depression (OR: 1.13; 95% CI: 1.06-1.19; *P* < 0.001), and poor physical health (OR: 1.14; 95% CI: 1.08-1.21; *P* < 0.001) were associated with higher odds of reporting high cholesterol. Additionally, being unable to afford a doctor (OR: 0.94; 95% CI: 0.90-0.99; *P* < 0.001) was associated with lower odds of having cholesterol checked among transgender individuals.

Our study, after propensity score matching, found that compared to cisgender individuals, transgender individuals were more likely to report having high cholesterol and recent cholesterol checks but were less likely to report taking cholesterol medications. The higher odds of high cholesterol levels in transgender individuals could be due in part to gender-affirming hormone therapies, as sex steroids may increase low-density lipoprotein cholesterol levels, or it could be due to social determinants of health.^4^^,^^5^ Despite having high cholesterol levels, transgender adults report taking fewer cholesterol medications. This may be attributed to various psychosocial stressors, including lower socioeconomic status, limited education, and difficulty accessing health care due to high costs. Additionally, the American Heart Association highlights the need for individualized risk assessment in transgender individuals, as current risk calculators do not account for gender identity or the effects of gender-affirming hormone therapy.^5^

Lack of insurance and poorer physical health further contribute to a higher prevalence of elevated cholesterol. Further stressors that can contribute to the poor physical and mental health outcomes include experiences of gender nonaffirmation, such as being misgendered, as well as stigma (including internalized stigma), rejection, discrimination, anticipatory anxiety, heightened vigilance, and the need to conceal one’s identity.^5^ One limitation of our study is that the data were self-reported by the participants, which means we cannot confirm actual lipid values or medication adherence. This method is susceptible to recall bias and cannot establish causality. Additionally, in some cases, the effect size (with ORs close to 1.00) may have limited clinical significance.

In sum, our findings highlight gaps in cholesterol management among transgender individuals that should be addressed to optimize the cardiovascular health of all persons for ASCVD prevention.

## Funding support and author disclosures

Dr Michos reports advisory board participation for Arrowhead, Bayer, Boehringer Ingelheim, Edwards Lifesciences, Ionis, Lilly, Merck, New Amsterdam, Novartis, Novo Nordisk, and Pfizer. Dr Michos is supported by the Amato Fund for Women’s Cardiovascular Health research at Johns Hopkins University, as well as an American Heart Association grant 946222 All other authors have reported that they have no relationships relevant to the contents of this paper to disclose.
